# Mechanism of tanshinones and phenolic acids from Danshen in the treatment of coronary heart disease based on co-expression network

**DOI:** 10.1186/s12906-019-2712-4

**Published:** 2020-02-03

**Authors:** Dongxue Wu, Mengqi Huo, Xi Chen, Yanling Zhang, Yanjiang Qiao

**Affiliations:** Beijing University of Chinese Medicine, State Administration of Traditional Chinese Medicine, Research Center of TCM-Information Engineering, Beijing, 100102 China

**Keywords:** Coronary heart disease, Danshen, Co-expression network

## Abstract

**Background:**

The tanshinones and phenolic acids in *Salvia miltiorrhiza* (also named Danshen) have been confirmed for the treatment of coronary heart disease (CHD), but the action mechanisms remain elusive.

**Methods:**

In the current study, the co-expression protein interaction network (Ce-PIN) was used to illustrate the differences between the tanshinones and phenolic acids of Danshen in the treatment of CHD. By integrating the gene expression profile data and protein-protein interactions (PPIs) data, the Ce-PINs of tanshinones and phenolic acids were constructed. Then, the Ce-PINs were analyzed by gene ontology enrichment analyzed based on the optimal algorithm.

**Results:**

It turned out that Danshen is able to treat CHD by regulating the blood circulation, immune response and lipid metabolism. However, phenolic acids may regulate the blood circulation by Extracellular calcium-sensing receptor (CaSR), Endothelin-1 receptor (EDNRA), Endothelin-1 receptor (EDNRB), Kininogen-1 (KNG1), tanshinones may regulate the blood circulation by Guanylate cyclase soluble subunit alpha-1 (GUCY1A3) and Guanylate cyclase soluble subunit beta-1 (GUCY1B3). In addition, both the phenolic acids and tanshinones may regulate the immune response or inflammation by T-cell surface glycoprotein CD4 (CD4), Receptor-type tyrosine-protein phosphatase C (PTPRC).

**Conclusion:**

Through the same targets of the same biological process and different targets of the same biological process, the tanshinones and phenolic acids synergistically treat coronary heart disease.

## Background

CHD is one of the ten leading diseases all over the world [1]. According to the World Health Organization, Coronary heart disease causes more than 700,000 deaths each year in China [2], which involves in thrombus formations [3], inflammation process [4], myocardial ischemia [5], etc. Traditional Chinese medicine (TCM) can treat the CHD by the synergistic effect of various components at a systemic level [6, 7]. Especially the blood stasis medicine, has been used for treating the blood stasis, obstruction of qi in the chest and cardialgia for thousands of year. Nevertheless, the mechanism of TCM for CHD is elaborated incompletely at a molecular level.

Danshen is the dry roots and rhizomes of *Salvia miltiorrhiza* Bge., which has the function of activating circulation and dispersing stasis. It has been widely used in clinics for the treatment of cardiovascular disease for hundreds of year [8–10]. The active ingredients of Danshen include water-soluble phenolic compounds such as salvianolic acid A, salvianolic acid B, together with lipophilic quinines compounds including tanshinone I, tanshinone IIA, cryptotanshinone [11–13]. Pharmacological experiments have been shown that both tanshinones and phenolic acids of Danshen have antioxidant, anti-inflammatory, inhibition of platelet aggregation, anti-thrombosis and so on [14–17]. However, the study of the difference between the tanshinones and phenolic acids of Danshen focused on specific activities, such as antimicrobial activity [18]. In this study, we will illustrate the holistic difference between tanshinones and phenolic acids for the treatment of coronary heart disease at the molecular level.

Proteins are vital macromolecules, which interact with each other to induce the biological function in an organism. Therefore, protein interaction network (PIN) could provide the basis of understanding cellular organization and processes. Recently, application of high-throughput technologies produces a large amount of protein interaction data, but the interactions cannot be directly used to identify signaling pathways or active networks [19]. The high-throughput sequencing analysis of gene expression has become increasingly valued as a promising tool for analyzing the molecular mechanism of formulas and the discovery of new anticancer targeted drugs [20, 21]. Thus, the combination of gene expression profiles and protein network information is more likely to be correct than date source alone for illustrating the mechanisms underlying the observed changes in activity of a biological process [22]. The PIN integrated with gene expression profile called the co-expression protein interaction network (Ce-PIN) has been used in the identification of a hub protein [23], elaborating the molecular mechanism of cancer [24] . And Ce-PIN has not been used in the study of tanshinones and phenolic acids mechanism for the treatment of other diseases.

In this study, the PINs were integrated with CHD gene expression profile from GEO to constructed the Ce-PIN; afterwards the Ce-PINs were analyzed by gene ontology enrichment analyzed based on the optimal algorithm. This research aimed at providing a novel approach to study treatment mechanisms of Danshen systematically and compare the difference between tanshinones and phenolic acids in treatment for CHD.

## Methods

### Data mining

The main active components of Danshen will be used to study the mechanism of Danshen in treating coronary heart disease. By literature retrieval, the main active components of Danshen were selected based on the principles that components are the main efficacy compounds and has the effect of treating CHD. Hence, salvianolic acid A, salvianolic acid B, protocatechuic aldehyde were chosen as the representative component of water-soluble compounds, and tanshinone I, tanshinone IIA, cryptotanshinone, dihydrotanshinone I were chosen as the representative compound of liposoluble compounds to study the mechanism of Danshen in treating coronary heart disease.

The target’s information of active components was obtained based on pharmacophore virtual screening and the component-protein interaction database including ChEMBL (https://www.ebi.ac.uk/chembl/#) and STITCH4.0 (http://stitch.embl.de/). The pharmacophore virtual screening is a method to search for pharmacophore that matches the active compounds based on the 100 pharmacophore models associated with blood circulation. The target, whose Fit value score with active components of Danshen above 0.7 was selected as the target of active components of Danshen. ChEMBL [25] is an Open Data database that allows users to search for components and targets, containing 5.4 million bioactivity measurements for more than 1 million compounds and 5200 protein targets. STITCH [26] is a database of protein-chemical interactions in which every interaction has a confidence score, and the confidence score above 0.7 was selected.

### Network construction

The protein-protein interaction information was extracted from the online updated databases of STRING 10(http://string-db.org). STRING [27] is a database of known and predicted protein-protein interactions, including direct (physical) and indirect (functional) associations. Every protein-protein interaction (PPI) has a confidence score, and confidence score above 0.7 were selected in order to ensure data reliability. The PPIs obtained from STRING 9.1 were imported into Cytoscape platform2.8.3, then the duplicated edges and self-loop edges were removed after union calculation using the Advanced Network Merge plug. The gene expression profile of CHD (No. GSE42148) was obtained from GEO (https://www.ncbi.nlm.nih.gov/geo/), including 13 disease samples. Then, the PPI network and CHD gene expression profile were integrated to construct the Ce-PIN.

### Network analysis

In this study, FAG-EC algorithm was used to cluster the co-expression networks under disease state. FAG-EC [28] algorithm is a fast hierarchical agglomerative algorithm based on edge clustering coefficients, which can deal with large complex networks due to the low computational complexity. The Complex Size Threshold is set to 4 to identify module contained at least four nodes. Based on the identified modules, GO enrichment analysis was used to predict possible biological roles of the modules by evaluating the involved biological processes, using the BinGO [29] plugin for Cytoscape platform whose significant selection was set to 0.05. Gene Ontology information and annotation information are from Gene ontology [30] (http://geneontology.org/).

## Results

### Data mining

The phenolic acids received a total of 42 targets (Additional file 1: Table S1). The tanshinones received a total of 101 targets. (Additional file 2: Table S2).

### Network construction

**Fig. 1:**
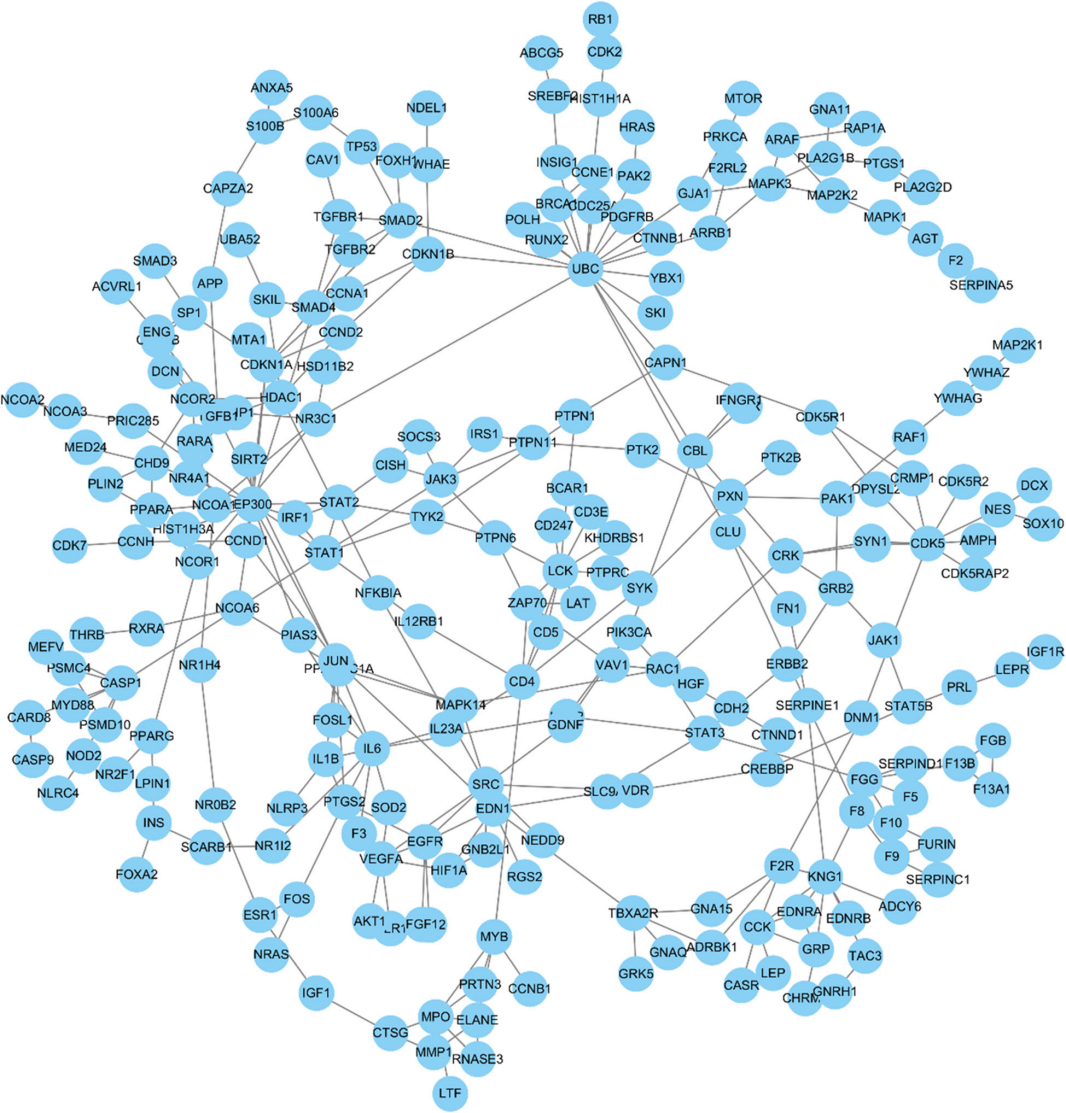
The subnetwork of phenolic acids

**Fig. 2:**
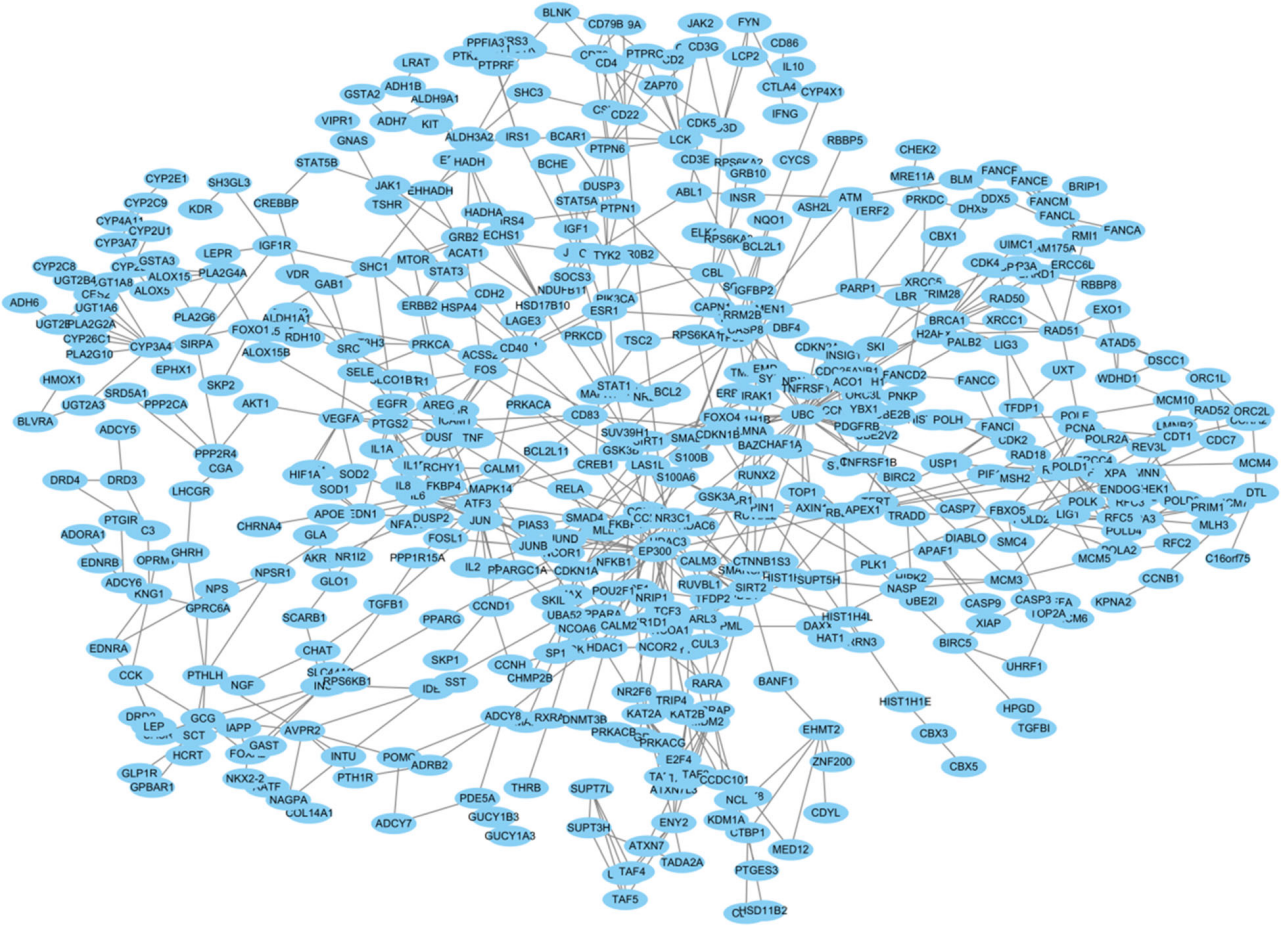
The subnetwork of tanshinones

The Ce-PIN of phenolic acids has 324 nodes and 399 edges; The Ce-PIN of tanshinones has 612 nodes and 891 edges. However, the constructed network was not fully connected due to the unclear human protein interactions at present, thus the largest subgraph network was selected to carry out subsequently by removing the duplicated edges and self-loop edges. The subnetwork of phenolic acids has 244 nodes and 338 edges (Fig. 1); the subnetwork of tanshinones has 520 nodes and 819 edges (Fig. 2).

## Discussion

### The Ce-PIN of phenolic acids

**Fig. 3:**
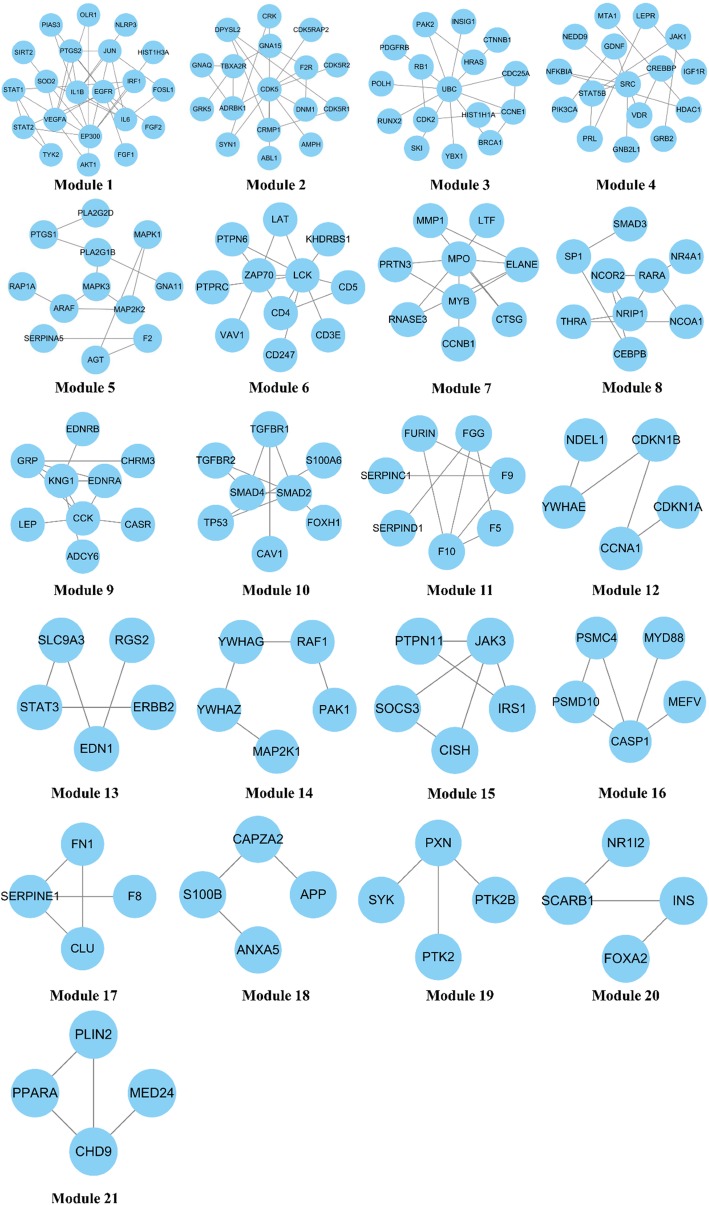
Modules in the Ce-PIN of phenolic acids

With the FAG-EC algorithm, 21 modules were identified (Fig. 3). All 21 modules included 244 of the total 324 proteins. Based on the identified modules, GO enrichment analysis was used to predict possible biological roles of the modules using the BinGO plugin for Cytoscape platform. For each module, the most significant GO biological processes were chosen to evaluate the mechanism of modules. The results have been shown in Table 1.

**Table 1:** GO biological process terms of phenolic acids modules display partially

Modules	GO terms	*P*-value
Module1	response to chemical	1.48 × 10^− 15^
Module2	blood coagulation	8.33 × 10^−9^
	coagulation	8.33 × 10^−9^
Module3	positive regulation of nucleobase-containing compound metabolic process	6.92 × 10^−11^
Module4	enzyme linked receptor protein signaling pathway	3.49 × 10^− 10^
Module5	positive regulation of protein phosphorylation	7.36 × 10^− 11^
Module6	regulation of T cell activation	4.49 × 10^−19^
	positive regulation of T cell activation	6.19 × 10^−18^
Module7	extracellular matrix disassembly	7.25 × 10^−8^
Module8	transcription initiation from RNA polymerase II promoter	8.57 × 10^−12^
Module9	blood circulation	2.17 × 10^−8^
	vasodilation	2.04 × 10^−7^
Module10	transforming growth factor beta receptor signaling pathway	5.35 × 10^−13^
Module11	blood coagulation	1.87 × 10^−10^
	coagulation	1.87 × 10^−10^
Module12	mitotic cell cycle	9.46 × 10^−9^
Module13	regulation of cardiac muscle hypertrophy	4.10 × 10^−6^
Module14	regulation of protein kinase activity	2.65 × 10^−6^
Module15	JAK-STAT cascade involved in growth hormone signaling pathway	2.09 × 10^−12^
Module16	immune system process	5.75 × 10^−6^
Module17	positive regulation of molecular function	2.01 × 10^−7^
Module18	blood coagulation	2.13 × 10^−5^
	coagulation	2.13 × 10^− 5^
Module19	signal complex assembly	4.78 × 10^−11^
Module20	positive regulation of lipid biosynthetic process	2.21 × 10^−5^
Module21	fatty acid transport	2.06 × 10^−5^
	cellular lipid metabolic process	2.92 × 10^−4^

The regulation of blood coagulation (Module 2、Module11、Module18) contained proteins such as proteinase-activated receptor 1 (F2R), thromboxane A2 receptor (TBXA2R), coagulation factor IX(F9), coagulation factor X(F10), coagulation factor V(F5), antithrombin-III (SERPINC1), heparin cofactor 2(SERPIND1), annexin A5(ANXA5). F2R is a member of the Proteinase-activated receptor which play critical roles in atherosclerosis [31]. It has been recognized as a candidate to remedy the patients with acute coronary syndromes [32]. Thromboxane A_2_(TXA_2_) is the arachidonate metabolite [33]. TBXA_2_R can interact specificity with TXA_2_ to induce platelet aggregation and constrict smooth muscle which belongs to the G-protein-coupled receptors superfamily [34]. A lot of TXA_2_ receptor antagonists have been developed as therapeutic antagonist for thrombosis, hypertension and asthma [35]. F9 is a vitamin K-dependent plasma protein involved in the intrinsic pathway of the coagulation cascade [36]. F9 inhibitors can attenuate clot formation as safe and effective anticoagulants [37]. F10 is a vitamin K-dependent plasma glycoprotein which is involved in the activation of thrombin by intrinsic and extrinsic pathway of Coagulation cascades [38]. F5 is essential cofactor in the prothrombinase complex and results in generation of thrombin [39, 40]. SERPINC1 can inhibit factor Xa and thrombin as a potent inhibitor of blood coagulation [41]. SERPIND1 is a plasma glycoprotein that inhibit thrombin specifically, which is activated by heparin or dermatan sulfate [42, 43]. ANXA5 is a member of the annexin proteins as anticoagulant and antithrombotic protein [44]. It has been demonstrated to inhibit production of thrombin and activation of factor X [45]. Previous study had demonstrated that Salvianolic acid A inhibited human platelet aggregation induced by ADP in vitro or vivo [15]. These indicate that water-soluble compounds extracted form Danshen may regulate blood coagulation in coronary atherosclerotic heart disease by F2R, TBXA2R, F9, F10, F5, SERPINC1, SERPIND1 and ANXA5.

The regulation of blood circulation (module 9) contained proteins such as extracellular calcium-sensing receptor (CASR), endothelin-1 receptor (EDNRA), endothelin b receptor (EDNRB), kininogen-1 (KNG1). CASR is a G protein-coupled transmembrane receptor [46], which maintain and regulate systemic calcium homeostasis by inhibits secretion of parathyroid hormone in parathyroid glands [47]. Previous study has suggested that CASR contracted vessel by mediating regulation of contraction in vascular smooth muscle cells [48]. The endothlin-1(ET-1) is a member of the endothelin family of peptides (ET-1, ET-2, ET-3), which can be mediated by ET_A_ and ET_B_ receptor. The ET_B_ receptor is non-specific receptor for ET-1, ET-2, ET-3, whereas The ET_A_ receptor has a higher affinity for ET-1 [49]. Binding of ET to ET receptors on the endothelium induce the production of NO and prostacyclin, which result in vasodilation [50]. KNG1 is cleaved by plasma prekallikrein in the kallikrein-kinin system, releasing bradykinin, a most potent vasodilator, resulting in the vasodilation [51, 52]. It is reported that Salvianolic acid B exerts vasodilation activity through NO related signals [53]. To sum up, water-soluble compounds of Danshen may play a role in blood circulation by CASR, EDNRA, EDNRB, KNG1, to treat the coronary heart disease.

The regulation of lipid metabolism (module 20, 21) contained proteins such as insulin (INS), peroxisome proliferator-activated receptor alpha (PPARA). INS promotes the synthesis of lipids, and inhibits their degradation, which is correlated with the increase of transcription factor steroid regulatory element-binding protein (SREBP)-1c [54]. Insulin resistance is a pathogenetic factor in the development of atherosclerosis in diabetes [55]. Recent studies have demonstrated that impaired insulin signaling accelerates atherosclerosis [56]. PPARA is a ligand-activated transcription factor, which expression by macrophages has antiatherogenic effects by modulation of cell cholesterol trafficking and inflammatory activity [57]. It is reported that salvianolic acids are the major effective components of DanQi pill in improving lipid metabolism in ischemic heart model, which may be mediated by regulating transcriptional factors such as PPARs, RXRA and PGC-1 alpha [58]. These indicate that water-soluble compounds extracted form Danshen may regulate lipid metabolism in coronary atherosclerotic heart disease by INS, PPARA.

The regulation of immune response (module 6, 16) contained proteins such as T-cell surface glycoprotein CD4(CD4), receptor-type tyrosine-protein phosphatase C(PTPRC), T-cell surface glycoprotein CD5(CD5), tyrosine-protein kinase lck (LCK), linker for activation of T-cells family member 1(LAT), caspase-1(CASP1). CD4 is cell surface glycoproteins expressed on T lymphocytes that play a major role in both the activation of mature peripheral T cells and the thymic differentiation process [59]. The down-regulation of CD5 may reduce the dysregulation proliferation of CD8^+^ T-cells. It has been reported that the water-soluble phenolic compound of Danshen were able to increase CD4 T cell to treat atherosclerosis by modulating the inflammation [60]. PTPRC is a member of protein tyrosine phosphatase (PTP) family that plays important role in immune response [61]. The LCK in the inactive state is modulated by RhoH contributing to the regulation of both ore-TCR and TCR signaling during T-cell development [62]. The LAT is a palmitoylated integral membrane adaptor protein that is phosphorylated by protein tyrosine kinases (PTK) and binds to the adaptors Gads, Grb2 and phospholipase Cγ1 (PLCγ1) to drive T-cell activation [63]. The CASP1 is a cysteine protease that acts as an essential regulator of inflammatory responses [64]. Innate immunity plays a role in both thrombosis and inflammation [65]. Recent research has reported that mast cells are participated in atherosclerosis by releasing proinflammatory molecules and vasoactive mediators [66]. It is reported that Salvianolic acid A blocked inflammatory responses by impairing NF-kappa B signaling [67]. These indicate that water-soluble compounds extracted form Danshen may regulate immune response/inflammation in coronary atherosclerotic heart disease by CD4, CD5, PTPRC, LCK, LAT, CASP1.

### The Ce-PIN of tanshinones

**Fig. 4:**
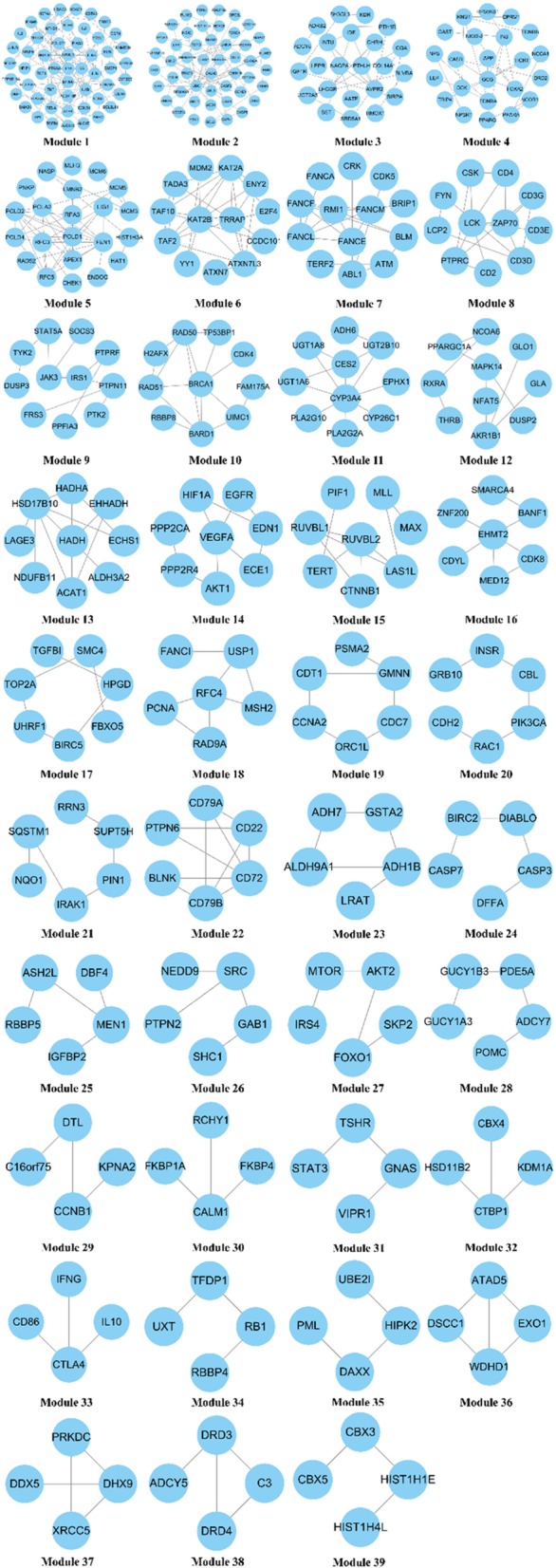
Modules in the Ce-PIN of tanshinones

With the FAG-EC algorithm, 39 modules were identified (Fig. 4). All 21 modules included 520 of the total 612 proteins. Based on the identified modules, GO enrichment analysis was used to predict possible biological roles of the modules using the BinGO plugin for Cytoscape platform. For each module, the most significant GO biological processes were chosen to evaluate the mechanism of modules. The results have been shown in Table 2.

**Table 2:** GO biological process terms of tanshinones modules display partially

Modules	GO terms	*P*-value
Module 1	response to chemical stimulus	1.64 × 10^−25^
Module 2	regulation of cellular protein metabolic process	5.07 × 10^−15^
Module 3	G-protein coupled receptor signaling pathway, coupled to cyclic nucleotide second messenger	3.86 × 10^−11^
Module 4	positive regulation of cellular process	1.23 × 10^−14^
Module 5	DNA strand elongation involved in DNA replication	9.21 × 10^−31^
Module 6	histone deubiquitination	1.25 × 10^−17^
Module 7	DNA metabolic process	3.76 × 10^−13^
Module 8	T cell receptor signaling pathway	4.80 × 10^−24^
	regulation of T cell activation	4.77 × 10^− 19^
Module 9	growth hormone receptor signaling pathway	1.65 × 10^−13^
Module 10	double-strand break repair	7.40 × 10^− 18^
Module 11	lipid metabolic process	6.71 × 10^− 6^
Module 12	transcription from RNA polymerase II promoter	2.63 × 10^−7^
Module 13	lipid oxidation	6.42 × 10^−9^
Module 14	positive regulation of catalytic activity	7.76 × 10^−10^
Module 15	histone acetylation	1.91 × 10^−6^
Module 16	nucleic acid metabolic process	4.33 × 10^−6^
Module 17	cell division	2.83 × 10^− 6^
Module 18	DNA repair	2.79 × 10^−10^
Module 19	M/G1 transition of mitotic cell cycle	6.21 × 10^−13^
Module 20	enzyme linked receptor protein signaling pathway	2.07 × 10^−7^
Module 21	positive regulation of cellular process	1.46 × 10^−5^
Module 22	positive regulation of immune response	1.37 × 10^−4^
	activation of immune response	7.48 × 10^−5^
Module 23	ethanol oxidation	1.19 × 10^−6^
Module 24	cellular component disassembly involved in execution phase of apoptosis	2.33 × 10^−10^
Module 25	histone lysine methylation	3.01 × 10^−8^
Module 26	enzyme linked receptor protein signaling pathway	5.33 × 10^−6^
Module 27	regulation of fatty acid beta-oxidation	3.30 × 10^−6^
Module 28	blood circulation	1.38 × 10^−5^
	blood coagulation	5.26 × 10^−5^
Module 29	regulation of chromosome condensation	1.31 × 10^−4^
Module 30	regulation of lipoprotein lipase activity	8.15 × 10^−6^
Module 31	G-protein coupled receptor signaling pathway, coupled to cyclic nucleotide second messenger	6.95 × 10^−7^
Module 32	negative regulation of histone modification	6.83 × 10^−6^
Module 33	regulation of T cell activation	1.93 × 10^−8^
	regulation of immune response	7.63 × 10^−7^
Module 34	G1 phase of mitotic cell cycle	1.06 × 10^−5^
Module 35	virus-host interaction	4.31 × 10^−8^
Module 36	heterochromatin maintenance	1.31 × 10^−4^
Module 37	double-strand break repair via nonhomologous end joining	2.99 × 10^−6^
Module 38	G-protein coupled receptor signaling pathway	3.78 × 10^−7^
Module 39	chromatin organization	4.23 × 10^−5^

The regulation of blood circulation/coagulation (module 28) contained proteins such as guanylate cyclase soluble subunit alpha-3(GUCY1A3), guanylate cyclase soluble subunit beta-1(GUCY1B3). GUCY1A3 and GUCY1B3 encodes for the α1 subunit and β1 subunit of soluble guanylate cyclase (SGC), respectively. GUCY1A3 mutations increases risk for moyamoya disease, achalasia and hypertension [68]. The SGC is the central enzyme in the NO-cGMP signalling pathway to protect the heart from ischemia and reperfusion injury, which has been used in treatment of coronary heart disease for 100 years [69]. It is reported that Tanshinone IIA protects against myocardial ischemia reperfusion injury by activating the PI3K/Akt/mTOR signaling pathway [70]. Hence, tanshinones may regulate the blood circulation by GUCY1A3 and GUCY1B3 to protects against myocardial ischemia reperfusion injury.

The regulation of lipid metabolism (module 20, 21) contained proteins such as group 10 secretory phospholipase A2(PLA2G10), cytochrome P450 3A4(CYP3A4). PLA2G10 belongs to the phospholipase A2 family, which plays a important role in atherogenesis [71]. The fatty acid can be hydrolyse specifically by the A2 group of phospholipases (PLA2s) at the sn-2, or second carbon positions on the glycerol backbone of the phospholipids, and release fatty acid and lysophospholipid [72]. The animal and human studies suggest that high levels of secretory phospholipase A2(sPLA2) may be implicated in the initial and later stages of the development of the atherosclerotic plaque [73, 74]. Cytochrome P450 3A4 is a member of the cytochrome P450(CYP) family. It is reported that CYP-mediated eicosanoid metabolism is dysregulated in certain subsets of CHD patients, and demonstrate that biomarkers of CYP epoxygenase and soluble epoxide hydrolase, but not CYP-hydroxylase, metabolism are altered in stable CHD patients relative to healthy individuals [75]. The study indicated that Tanshinone IIA was able to regulate lipid metabolism by miR-33a/SREBP-2/Pcsk9 signaling pathway to reduce lipid deposition in the liver of hyperlipidemia rat [76].

The regulation of immune responsec(module 8, 22,33) contained proteins such as T-cell surface glycoprotein CD4(CD4), receptor-type tyrosine-protein phosphatase C(PTPRC), tyrosine-protein kinase Fyn(FYN), T-cell surface glycoprotein CD3 epsilon chain(CD3E), T-cell surface glycoprotein CD3 gamma chain(CD3G), T-cell surface glycoprotein CD3 delta chain(CD3D), B-cell antigen receptor complex-associated protein beta chain(CD79B), B-cell antigen receptor complex-associated protein alpha chain(CD79A). CD4 is cell surface glycoproteins expressed on T lymphocytes that play a major role in both the activation of mature peripheral T cells and the thymic differentiation process [77]. PTPRC is a member of protein tyrosine phosphatase (PTP) family that plays a important role in immune response [60]. The LCK and FYN are two members of the Src family of tyrosine kinases which play important role in the αβTCR-coupled signaling pathway. The expression level’s change of Lck and Fyn induce T cell development and maturation [78]. The CD3E, CD3G, CD3D are the components of TCR/CD3 complex which are expressed on the cell surface to mediates signal transduction [79, 80]. The CD79A and CD70B are transmembrane glycoproteins belonging to the Ig superfamily. They play an immunomodulatory role by mediating surface expression and signaling of diverse B cell receptor complexes on precursor, immature, and mature B cells [81]. Innate immunity plays a role in both thrombosis and inflammation [64]. Dendritic cells (DC), the potent antigen-presenting cells, stimulate T-cell proliferation and activation to induce the growth of atherosclerotic plaques during adaptive immunity [82]. Tanshinone IIA had been shown to inhibit DC maturation and decreases the expression of proinflammatory cytokines to decrease the growth of atherosclerotic lesions [83]. These indicate that liposoluble compounds extracted form Danshen may regulate immune response/inflammation in coronary atherosclerotic heart disease by CD4, PTPRC, FYN, CD3E, CD3G, CD3D, CD79B, CD79A.

### The synergetic effects of Danshen

**Fig. 5:**
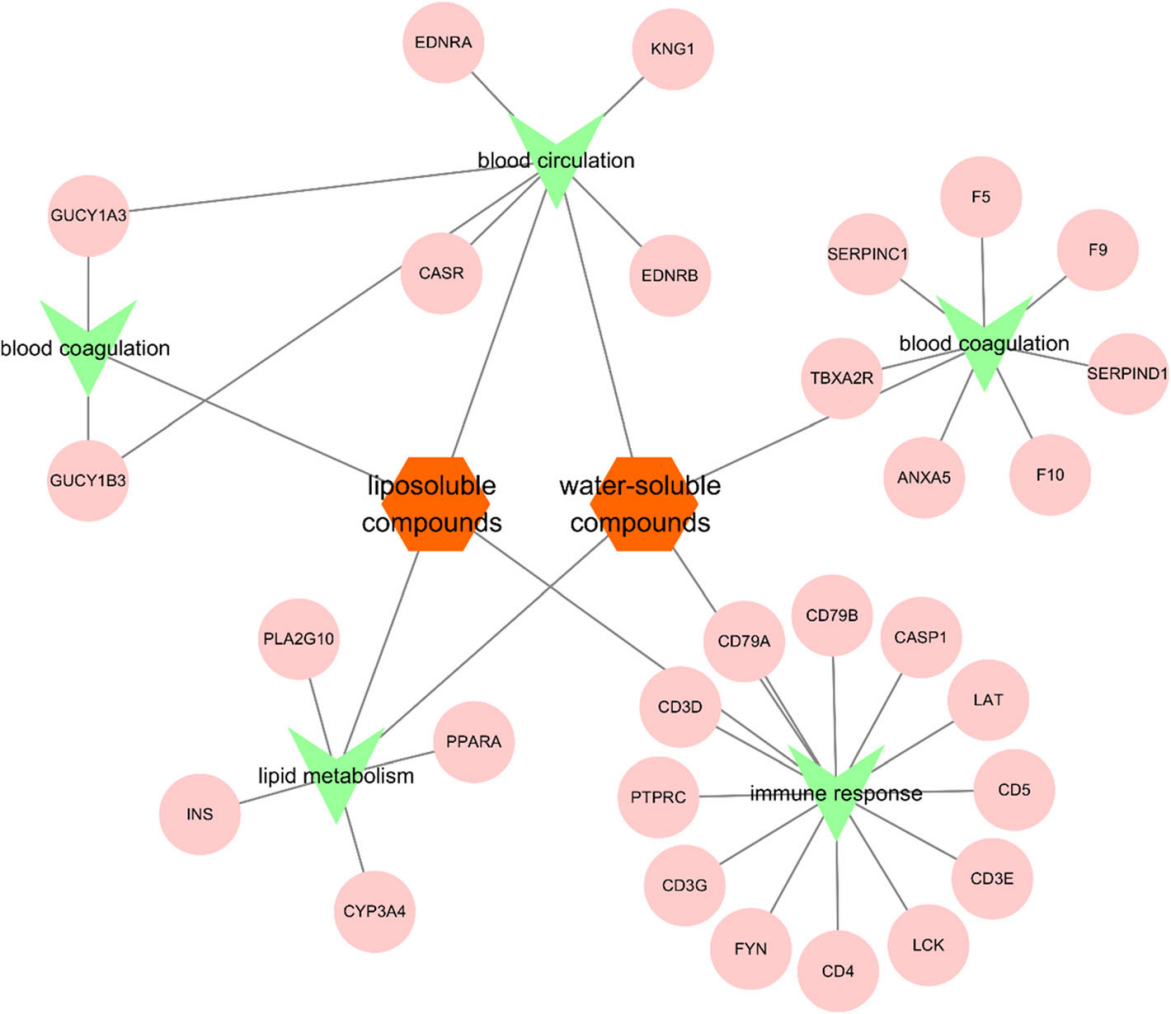
The Synergetic Effects of Danshen

According to the analysis of FAG-EC algorithm and GO enrichment, Danshen may play the role in the treatment of CHD by regulating the blood circulation, immune response and lipid metabolism. However, phenolic acids may regulate the blood circulation by CASR, EDNRA, EDNRB, KNG1, tanshinones regulated the blood circulation by GUCY1A3 and GUCY1B3 (Fig. 5). It is suggested that two compounds of Danshen may regulate the same metabolic process via the different module mediated by different proteins. In addition, both the phenolic acids and tanshinones may regulate the immune response or inflammation by CD4, PTPRC. It is suggested that two compounds of Danshen may regulate the same metabolic process via the different module mediated by the same proteins. This indicated that the synergy of phenolic acids and tanshinones was able to be illustrated based on the functional modules.

## Conclusions

In this study, the Ce-PIN of phenolic acids and tanshinones were established to elaborate the mechanism of treatment of CHD based on module-based network analysis approach. The phenolic acids may be partly attributed to the regulation of blood coagulation/circulation process, immune/inflammation process, lipid metabolic process to treat CHD, while the tanshinones may treat CHD through the regulation of blood circulation process, immune/inflammation process, lipid metabolic process. The two compounds of Danshen may regulate the same metabolic pathway through different modules, which reflect the synergistic effect between Chinese medicine components at the molecular level. It would be helpful for guiding the research and development of the novel drugs of Danshen. Further experiments are needed to confirm the conclusions. This study provides a novel approach to understand the mechanisms of Danshen of treatment in CHD. What’s more, the scientific intension of “synergy” of TCM may be also illustrated based on the functional modules at the molecular level.

## Supplementary information

**Additional file 1: Table S1.** The targets’ information of phenolic acids.

**Additional file 2: Table S2.** The targets’ information of tanshinones.

## Data Availability

The datasets generated and analyzed during the study are available from the corresponding author on reasonable request.

## Abbreviations

ANXA5: Annexin A5
CASP1: Caspase-1
CASR: Extracellular calcium-sensing receptor
CD3D: T-cell surface glycoprotein CD3 delta chain
CD3E: T-cell surface glycoprotein CD3 epsilon chain
CD3G: T-cell surface glycoprotein CD3 gamma chain
CD4: T-cell surface glycoprotein CD4
CD5: T-cell surface glycoprotein CD5
CD79A: B-cell antigen receptor complex-associated protein alpha chain
CD79B: B-cell antigen receptor complex-associated protein beta chain
Ce-PIN: Co-expression protein interaction networks
CYP3A4: Cytochrome P450 3A4
EDNRA: Endothelin-1 receptor
EDNRB: Endothelin b receptor
F10: Coagulation factor X
F2R: Proteinase-activated receptor 1
F5: Coagulation factor V
F9: Coagulation factor IX
FYN: Tyrosine-protein kinase Fyn
GUCY1A3: Guanylate cyclase soluble subunit alpha-3
GUCY1B3: Guanylate cyclase soluble subunit beta-1
INS: insulin
KNG1: Kininogen-1
LAT: Linker for activation of T-cells family member 1
LCK: Tyrosine-protein kinase lck
PIN: Protein interaction network
PLA2G10: Group 10 secretory phospholipase A2
PPARA: Peroxisome proliferator-activated receptor alpha
PPI: Protein-protein interaction
PTPRC: Receptor-type tyrosine-protein phosphatase C
SERPINC1: Antithrombin-III
SERPIND1: Heparin cofactor 2
TBXA2R: Thromboxane A2 receptor
TCM: Traditional Chinese medicine
