# Supplementary material for: Mechanism of tanshinones and phenolic acids from Danshen in the treatment of coronary heart disease based on co-expression network
Source: BMC Complement Med Ther. 2020 Feb 3;20:28. doi: 10.1186/s12906-019-2712-4 (PMC7076864; doi:10.1186/s12906-019-2712-4)
Supplement: Supplementary file 1 — Additional file 1: Table S1. The targets’ information of phenolic acids. [file 12906_2019_2712_MOESM1_ESM.docx]

**Mechanism of tanshinones and phenolic acids from Danshen in** **the Treatment of Coronary heart disease**

**based on** **Co-expression Network**

**Dongxue Wu^[[1]](#footnote-1)^, Mengqi Huo^1^, Xi Chen^1^, Yanling Zhang^1,^^[[2]](#footnote-2)^* and Yanjiang Qiao^1,^***

Table S1. The targets’ information of phenolic acids

| Uniprot ID | Gene Name | Protein Name | Source |
| --- | --- | --- | --- |
| P06239 | LCK | Tyrosine-protein kinase Lck | ChEMBL |
| P12931 | SRC | Proto-oncogene tyrosine-protein kinase Src | ChEMBL |
| P42224 | STAT1 | Signal transducer and activator of transcription 1-alpha/beta | ChEMBL |
| P51692 | STAT5B | Signal transducer and activator of transcription 5B | ChEMBL |
| P40763 | STAT3 | Signal transducer and activator of transcription 3 | ChEMBL |
| O94925 | GLS | Glutaminase kidney isoform, mitochondrial | ChEMBL |
| Q9UNA4 | POLI | DNA polymerase iota | ChEMBL |
| Q96KQ7 | EHMT2 | Histone-lysine N-methyltransferase EHMT2 | ChEMBL |
| B2RXH2 | KDM4E | Lysine-specific demethylase 4E | ChEMBL |
| O75164 | KDM4A | Lysine-specific demethylase 4A | ChEMBL |
| Q9UIF8 | BAZ2B | Bromodomain adjacent to zinc finger domain protein 2B | ChEMBL |
| P51151 | RAB9A | Ras-related protein Rab-9A | ChEMBL |
| Q06278 | AOX1 | Aldehyde oxidase | STITCH |
| P09601 | HMOX1 | Heme oxygenase 1 | STITCH |
| P55157 | MTTP | Microsomal triglyceride transfer protein large subunit | pharmacophore |
| P08235 | NR3C2 | Mineralocorticoid receptor | pharmacophore |
| P00742 | F10 | Coagulation factor X | pharmacophore |
| O75469 | NR1I2 | Nuclear receptor subfamily 1 group I member 2 | pharmacophore |
| Q07869 | PPARA | Peroxisome proliferator-activated receptor alpha | pharmacophore |
| P25101 | EDNRA | P25101 | pharmacophore |
| P24941 | CDK2 | Cyclin-dependent kinase 2 | pharmacophore |
| P08684 | CYP3A4 | P08684 | pharmacophore |
| P05164 | MPO | Myeloperoxidase | pharmacophore |
| P18031 | PTPN1 | Tyrosine-protein phosphatase non-receptor type 1 | pharmacophore |
| Q00535 | CDK5 | Cyclin-dependent-like kinase 5 | pharmacophore |
| P00734 | F2 | Prothrombin | pharmacophore |
| P19793 | RXRA | Retinoic acid receptor RXR-alpha | pharmacophore |
| O76074 | PDE5A | cGMP-specific 3',5'-cyclic phosphodiesterase | pharmacophore |
| P04035 | HMGCR | 3-hydroxy-3-methylglutaryl-coenzyme A reductase | pharmacophore |
| P30556 | AGTR1 | Type-1 angiotensin II receptor | pharmacophore |
| P09917 | ALOX5 | Arachidonate 5-lipoxygenase | pharmacophore |
| P12821 | ACE | Angiotensin-converting enzyme | pharmacophore |
| P35354 | PTGS2 | Prostaglandin G/H synthase 2 | pharmacophore |
| Q9UHC9 | NPC1L1 | NPC1-like intracellular cholesterol transporter 1 | pharmacophore |
| P24530 | EDNRB | Endothelin B receptor | pharmacophore |
| P29466 | CASP1 | Caspase-1 | pharmacophore |
| P03372 | ESR1 | Estrogen receptor | pharmacophore |
| P15056 | BRAF | Serine/threonine-protein kinase B-raf | pharmacophore |
| Q15796 | SMAD2 | Mothers against decapentaplegic homolog 2 | Chemprot |
| P01137 | TGFB1 | Retinoic acid receptor RXR-alpha | Chemprot |
| P04271 | S100B | cGMP-specific 3',5'-cyclic phosphodiesterase | Chemprot |
| P84022 | SMAD3 | Mothers against decapentaplegic homolog 3 | Chemprot |

1. * Correspondence: zhangyanling@bucm.edu.cn; yjqiao@bucm.edu.cn;

   1 Beijing University of Chinese Medicine, State Administration of Traditional Chinese Medicine, Research Center of TCM-Information Engineering, Beijing 100102, China; [↑](#footnote-ref-1)
2. [↑](#footnote-ref-2)
