# Supplementary material for: Mechanism of tanshinones and phenolic acids from Danshen in the treatment of coronary heart disease based on co-expression network
Source: BMC Complement Med Ther. 2020 Feb 3;20:28. doi: 10.1186/s12906-019-2712-4 (PMC7076864; doi:10.1186/s12906-019-2712-4)
Supplement: Supplementary file 2 — Additional file 2: Table S2. The targets’ information of tanshinones. [file 12906_2019_2712_MOESM2_ESM.docx]

Table S2. The targets’ information of tanshinones

| Uniprot ID | Gene Name | Protein Name | Source |
| --- | --- | --- | --- |
| P04637 | TP53 | Cellular tumor antigen p53 | BOTH |
| P40763 | STAT3 | Signal transducer and activator of transcription 3 | BOTH |
| Q9UNA4 | POLI | DNA polymerase iota | ChEMBL |
| P00352 | ALDH1A1 | Retinal dehydrogenase 1 | ChEMBL |
| P15428 | HPGD | 15-hydroxyprostaglandin dehydrogenase [NAD(+)] | ChEMBL |
| P06746 | POLB | DNA polymerase beta | ChEMBL |
| Q99714 | HSD17B10 | 3-hydroxyacyl-CoA dehydrogenase type-2 | ChEMBL |
| Q9Y253 | POLH | DNA polymerase eta | ChEMBL |
| P28482 | MAPK1 | Mitogen-activated protein kinase 1 | ChEMBL |
| Q9NUW8 | TDP1 | Tyrosyl-DNA phosphodiesterase 1 | ChEMBL |
| P39748 | FEN1 | Flap endonuclease 1 | ChEMBL |
| P22303 | ACHE | Acetylcholinesterase | ChEMBL |
| Q9UBT6 | POLK | DNA polymerase kappa | ChEMBL |
| P46063 | RECQL | ATP-dependent DNA helicase Q1 | ChEMBL |
| P18054 | ALOX12 | Arachidonate 12-lipoxygenase, 12S-type | ChEMBL |
| O94782 | USP1 | Ubiquitin carboxyl-terminal hydrolase 1 | ChEMBL |
| O14746 | TERT | Telomerase reverse transcriptase | ChEMBL |
| P15121 | AKR1B1 | Aldo-keto reductase family 1 member B1 | ChEMBL |
| P23141 | CES1 | Liver carboxylesterase 1 | ChEMBL |
| Q06124 | PTPN11 | Tyrosine-protein phosphatase non-receptor type 11 | ChEMBL |
| P29350 | PTPN6 | Tyrosine-protein phosphatase non-receptor type 6 | ChEMBL |
| O00748 | CES2 | Cocaine esterase | ChEMBL |
| O75496 | GMNN | Geminin | ChEMBL |
| Q96QE3 | ATAD5 | ATPase family AAA domain-containing protein 5 | ChEMBL |
| P10636 | MAPT | Microtubule-associated protein tau | ChEMBL |
| Q16236 | NFE2L2 | Nuclear factor erythroid 2-related factor 2 | ChEMBL |
| P84022 | SMAD3 | Mothers against decapentaplegic homolog 3 | ChEMBL |
| P02545 | LMNA | Prelamin-A/C | ChEMBL |
| Q9NR56 | MBNL1 | Muscleblind-like protein 1 | ChEMBL |
| P49798 | RGS4 | Regulator of G-protein signaling 4 | ChEMBL |
| O14727 | APAF1 | Apoptotic protease-activating factor 1 | ChEMBL |
| P83916 | CBX1 | Chromobox protein homolog 1 | ChEMBL |
| Q96KQ7 | EHMT2 | Histone-lysine N-methyltransferase EHMT2 | ChEMBL |
| Q9UIF8 | BAZ2B | Bromodomain adjacent to zinc finger domain protein 2B | ChEMBL |
| O75164 | KDM4A | Lysine-specific demethylase 4A | ChEMBL |
| B2RXH2 | KDM4E | Lysine-specific demethylase 4E | ChEMBL |
| Q9Y468 | L3MBTL1 | Lethal(3)malignant brain tumor-like protein 1 | ChEMBL |
| Q92830 | KAT2A | Histone acetyltransferase KAT2A | ChEMBL |
| P11473 | VDR | Vitamin D3 receptor | ChEMBL |
| P04150 | NR3C1 | Glucocorticoid receptor | ChEMBL |
| P10275 | AR | Androgen receptor | ChEMBL |
| P06401 | PGR | Progesterone receptor | ChEMBL |
| P63092 | GNAS | Guanine nucleotide-binding protein G(s) subunit alpha isoforms short | ChEMBL |
| Q13526 | PIN1 | Peptidyl-prolyl cis-trans isomerase NIMA-interacting 1 | ChEMBL |
| P38398 | BRCA1 | Breast cancer type 1 susceptibility protein | ChEMBL |
| P54132 | BLM | Bloom syndrome protein | ChEMBL |
| Q99700 | ATXN2 | Ataxin-2 | ChEMBL |
| Q13148 | TARDBP | TAR DNA-binding protein 43 | ChEMBL |
| P01215 | CGA | Glycoprotein hormones alpha chain | ChEMBL |
| P04637 | TP53 | Cellular tumor antigen p53 | ChEMBL |
| O75874 | IDH1 | Isocitrate dehydrogenase [NADP] cytoplasmic | ChEMBL |
| Q14191 | WRN | Werner syndrome ATP-dependent helicase | ChEMBL |
| P18031 | PTPN1 | Thyroid hormone receptor beta | ChEMBL |
| P08575 | PTPRC | Receptor-type tyrosine-protein phosphatase C | ChEMBL |
| P17706 | PTPN2 | Tyrosine-protein phosphatase non-receptor type 2 | ChEMBL |
| P43378 | PTPN9 | Tyrosine-protein phosphatase non-receptor type 9 | ChEMBL |
| Q03164 | KMT2A | Histone-lysine N-methyltransferase 2A | ChEMBL |
| P10828 | THRB | Thyroid hormone receptor beta | ChEMBL |
| P43220 | GLP1R | Glucagon-like peptide 1 receptor | ChEMBL |
| Q6W5P4 | NPSR1 | Neuropeptide S receptor | ChEMBL |
| P10586 | PTPRF | Receptor-type tyrosine-protein phosphatase F  GenePTPRF | ChEMBL |
| Q9Y6L6 | SLCO1B1 | Solute carrier organic anion transporter family member 1B1 | ChEMBL |
| Q9NPD5 | SLCO1B3 | Solute carrier organic anion transporter family member 1B3 | ChEMBL |
| C9J3U5 | DIF | Dorsal-related immunity factor Dif | STITCH |
| Q9BXM9 | FSD1L | FSD1-like protein | STITCH |
| P18847 | ATF3 | Cyclic AMP-dependent transcription factor ATF-3 | STITCH |
| P10145 | IL8 | Interleukin-8 | STITCH |
| P24385 | CCND1 | G1/S-specific cyclin-D1 | STITCH |
| Q9BTV5 | FSD1 | Fibronectin type III and SPRY domain-containing protein 1 | STITCH |
| P05305 | EDN1 | Endothelin-1 | STITCH |
| P60323 | NOS3 | Nanos homolog 3 | STITCH |
| O75469 | NR1I2 | Nuclear receptor subfamily 1 group I member 2 | STITCH |
| P42574 | CASP3 | Caspase-3 | STITCH |
| P35354 | PTGS2 | Prostaglandin G/H synthase 2 | STITCH |
| P01308 | INS | Insulin | STITCH |
| P06276 | BCHE | Cholinesterase | STITCH |
| O75469 | NR1I2 | Nuclear receptor subfamily 1 group I member 2 | STITCH |
| P35228 | NOS2 | Nitric oxide synthase, inducible | STITCH |
| P11712 | CYP2C9 | Cytochrome P450 2C9 | pharmacophore |
| P08684 | CYP3A4 | Cytochrome P450 3A4 | pharmacophore |
| P24941 | CDK2 | Cyclin-dependent kinase 2 | pharmacophore |
| P37231 | PPARG | Peroxisome proliferator-activated receptor gamma | pharmacophore |
| Q92830 | GCN5 | Histone acetyltransferase KAT2A | pharmacophore |
| O76074 | PDE5A | cGMP-specific 3',5'-cyclic phosphodiesterase | pharmacophore |
| P14061 | HSD17B1 | Estradiol 17-beta-dehydrogenase 1 | pharmacophore |
| P54132 | BLM | Bloom syndrome protein | Chemprot |
| P00352 | ALDH1A1 | Retinal dehydrogenase 1 | Chemprot |
| O94782 | USP1 | Ubiquitin carboxyl-terminal hydrolase 1 | Chemprot |
| P10636 | MAPT | Microtubule-associated protein tau | Chemprot |
| P46063 | RECQL | ATP-dependent DNA helicase Q1 | Chemprot |
| P04637 | TP53 | Cellular tumor antigen p53 | Chemprot |
| P15428 | HPGD | 15-hydroxyprostaglandin dehydrogenase [NAD(+)] | Chemprot |
| P02545 | LMNA | Prelamin-A/C | Chemprot |
| P10828 | THRB | Thyroid hormone receptor beta | Chemprot |
| P08684 | CYP3A4 | Cytochrome P450 3A4 | Chemprot |
| P28482 | MAPK1 | Mitogen-activated protein kinase 1 | Chemprot |
| Q99714 | HSD17B10 | 3-hydroxyacyl-CoA dehydrogenase type-2 | Chemprot |
| Q92830 | KAT2A | Histone acetyltransferase KAT2A | Chemprot |
| B2RXH2 | KDM4E | Lysine-specific demethylase 4E | Chemprot |
| Q03164 | KMT2A | Histone-lysine N-methyltransferase 2A | Chemprot |
| O00255 | MEN1 | Menin | Chemprot |
